# The *Aegilops ventricosa* 2N^v^S segment in bread wheat: cytology, genomics and breeding

**DOI:** 10.1007/s00122-020-03712-y

**Published:** 2020-11-12

**Authors:** Liangliang Gao, Dal-Hoe Koo, Philomin Juliana, Trevor Rife, Daljit Singh, Cristiano Lemes da Silva, Thomas Lux, Kevin M. Dorn, Marshall Clinesmith, Paula Silva, Xu Wang, Manuel Spannagl, Cecile Monat, Bernd Friebe, Burkhard Steuernagel, Gary J. Muehlbauer, Sean Walkowiak, Curtis Pozniak, Ravi Singh, Nils Stein, Martin Mascher, Allan Fritz, Jesse Poland

**Affiliations:** 1 Department of Plant Pathology and Wheat Genetics Resource Center, Kansas State University, 1712 Claflin Road, Manhattan, KS 66506 USA; 2 Global Wheat Program, International Maize and Wheat Improvement Center (CIMMYT), El Batan, 56237 Texcoco, CP Mexico; 3 Department of Agronomy, Kansas State University, 1712 Claflin Road, Manhattan, KS 66506 USA; 4 Plant Genome and Systems Biology (PGSB), Helmholtz Center Munich, Ingolstaedter Landstr. 1, 85764 Neuherberg, Germany; 5 Leibniz Institute of Plant Genetics and Crop Plant Research (IPK) Gatersleben, Corrensstr. 3, 06466 Seeland, Germany; 6 John Innes Centre, Computational and Systems Biology, Norwich Research Park, Norwich, NR47UH UK; 7 Department of Agronomy and Plant Genetics, University of Minnesota, 1991 Upper Buford Circle, 411 Borlaug Hall, Saint Paul, MN 55108 USA; 8 Crop Development Centre, University of Saskatchewan, Agriculture Building, 51 Campus Drive, Saskatoon, SK S7N 5A8 Canada; 9 Center for Integrated Breeding Research (CiBreed), Georg-August-University Göttingen, 37073 Göttingen, Germany; 10 German Centre for Integrative Biodiversity Research (iDiv) Halle-Jena-Leipzig, 04103 Leipzig, Germany; 11 Programa de Cultivos de Secano, Instituto Nacional de Investigación Agropecuaria (INIA), Estación Experimental La Estanzuela, Ruta 50, km 11.5, 70006 Colonia, Uruguay; 12 United States Department of Agriculture Agricultural Research Service, 1701 Centre Avenue, Fort Collins, CO 80526 USA; 13 Grain Research Laboratory, Canadian Grain Commission, Winnipeg, MB Canada

## Abstract

**Key message:**

The first cytological characterization of the 2N^v^S segment in hexaploid wheat; complete de novo assembly and annotation of 2N^v^S segment; 2N^v^S frequency is increasing 2N^v^S and is associated with higher yield.

**Abstract:**

The *Aegilops ventricosa* 2N^v^S translocation segment has been utilized in breeding disease-resistant wheat crops since the early 1990s. This segment is known to possess several important resistance genes against multiple wheat diseases including root knot nematode, stripe rust, leaf rust and stem rust. More recently, this segment has been associated with resistance to wheat blast, an emerging and devastating wheat disease in South America and Asia. To date, full characterization of the segment including its size, gene content and its association with grain yield is lacking. Here, we present a complete cytological and physical characterization of this agronomically important translocation in bread wheat. We de novo assembled the 2N^v^S segment in two wheat varieties, ‘Jagger’ and ‘CDC Stanley,’ and delineated the segment to be approximately 33 Mb. A total of 535 high-confidence genes were annotated within the 2N^v^S region, with > 10% belonging to the nucleotide-binding leucine-rich repeat (NLR) gene families. Identification of groups of NLR genes that are potentially N genome-specific and expressed in specific tissues can fast-track testing of candidate genes playing roles in various disease resistances. We also show the increasing frequency of 2N^v^S among spring and winter wheat breeding programs over two and a half decades, and the positive impact of 2N^v^S on wheat grain yield based on historical datasets. The significance of the 2N^v^S segment in wheat breeding due to resistance to multiple diseases and a positive impact on yield highlights the importance of understanding and characterizing the wheat pan-genome for better insights into molecular breeding for wheat improvement.

**Electronic supplementary material:**

The online version of this article (10.1007/s00122-020-03712-y) contains supplementary material, which is available to authorized users.

## Introduction

Bread wheat (*Triticum aestivum* L*.*), a crop that provides roughly one-fifth of the calories consumed by humans, has a very large genome of 16 gigabase (IWGSC 2018). In addition to its size, one remarkable feature of the allohexaploid (2*n* = 6*x* = 42, AABBDD) wheat genome is its high plasticity. The wheat genome can harbor, and easily tolerate, large structural variations such as whole or partial chromosome deletions, insertions and substitutions. The high plasticity of the genome likely contributed to the successful spread of bread wheat as a staple food crop around the globe. This high plasticity also readily enables introgression of novel alleles via chromosome translocations from related species (Friebe et al. 1996).

Wild wheat relatives are rich sources of genetic diversity for bread wheat. To date, over 52 Triticeae species across 13 genera have been successfully utilized to enrich the genetic diversity of cultivated wheat (Wulff and Moscou 2014). Among them, *Aegilops ventricosa* Tausch is a tetraploid (2*n* = 4*x* = 28, NNDD) species. A segment from *Ae*. *ventricosa* was first introduced into the wheat cultivar VPM1 by the French cytogeneticist G. Doussinault (1983) in an attempt to transfer a gene for eyespot (caused by the fungus *Pseudocercosporella herpotrichoides*) resistance onto chromosome 7D, and the same segment was later found to possess resistance to three rust diseases: leaf rust (caused by *Puccinia triticina*), stem rust (*Puccinia graminis*) and stripe rust (*Puccinia striiformis f.sp. tritici*) (Bariana and McIntosh 1994). Tanguy et al. (2005) demonstrated that there is a preexisting translocation of this same chromosome in *Ae. ventricosa* (6N^V^L-2N^v^S). Badaeva et al (2008) later detected a small pAs1 site at the tip of chromosome 2A in hexaploid wheat VPM1. The 2N^v^S segment was also found to possess resistance against nematode diseases (Jahier et al. 2001; Williamson et al. 2013) and, more recently, against the wheat blast fungus *Magnaporthe oryze Triticum* pathotype (MoT), the causal agent of the devastating and emerging wheat blast disease (Cruz et al. 2016; Cruz and Valent 2017). Furthermore, new reports suggest that this fragment is contributing to lodging resistance in bread wheat (Singh et al. 2019).

Breeders from various regions around the world have introduced the *Ae. ventricosa* 2N^v^S segment into their breeding programs. The hard winter wheat breeding program in Kansas (KS) has relied on 2N^v^S and the rust resistance provided through this segment since it was first introduced in the extensively cultivated variety ‘Jagger.’ Over time, the 2N^v^S segment has been introduced into other breeding programs in the US central plains. One means of tracking this introduction in the USA is via the utilization of the USDA Regional Performance Nursery (RPN), which evaluates near-release varieties from over 20 public and private wheat breeding programs over dozens of testing locations. Internationally, the CIMMYT spring bread wheat breeding program has focused on developing improved germplasm with broad adaptation, high yield potential and strong disease resistance. From pedigrees, it is known that the CIMMYT wheat breeding program has made extensive use of the 2N^v^S segment. These wheat breeding programs (KS and CIMMYT) and testing networks (USDA-RPN) provide an optimal platform to systematically study the 2N^v^S frequency changes over time and assess the impact of 2N^v^S on grain yield. An examination of the association of 2N^v^S and wheat grain yield in a wide germplasm collection across multiple breeding programs will enable us to study whether this 2N^v^S segment is associated with any yield penalty as disease resistance is often assumed to be costly (Brown 2002).

With the advancement of genomics technologies, multiple genomes for wild and domesticated wheat species have been assembled to reference-grade quality (Luo et al. 2017; Avni et al. 2017; IWGSC 2018; Ling et al. 2018). The hexaploid bread wheat genome is especially high in plasticity and contains a wide range of introgressions and substitutions. To gain a better picture of the wheat pan-genome, a consortium of scientists and breeders worked to generate reference quality wheat genomes for a global selection of wheat cultivars. The Jagger genome represents one of the Wheat 10 + Genomes and was assembled recently (Walkowiak et al. 2020). Jagger was an extremely popular variety which was grown in more than 12 nations and planted on over 10 million acres per year for almost a decade in the central USA alone. One possible feature that contributed to its success as a popular winter wheat variety is the 2N^v^S segment (Fang et al. 2011) and its associated disease and physiological benefits (Singh et al. 2019). It is likely that the 2N^v^S segment is also widely present in global wheat germplasms through germplasm contributions of the CIMMYT spring wheat breeding program, which would have a very a large impact on global wheat production. Thus, genome characterization of this agronomically important segment and the study of its association with crop yield is a necessary and relevant task.

The objectives of this study were to: (1) provide cytological evidence for the 2N^v^S segment present in bread wheat using genomic in situ hybridization (GISH); (2) physically assemble the segment to a reference-grade assembly; (3) characterize the 2N^v^S gene content and transcription patterns across different tissues and growth stages; (4) predict 2N^v^S presence in a variety of breeding programs based on historically available genotyping by sequencing (GBS) datasets; and (5) evaluate the 2N^v^S frequency and yield impact across years for KS, USDA-RPN and CIMMYT wheat breeding and testing programs.

## Materials and methods

### Plant materials

The winter bread wheat cultivar Jagger was used for de novo genome assembly and functional annotation of the *Ae. ventricosa* 2N^v^S alien segment. The spring wheat cultivar, CDC Stanley, was used for independent assembly and validation of the 2N^v^S segment assembly. Jagger and CDC Stanley are widely grown wheat varieties that were assembled to reference grade in the panel of the Wheat 10 + Genome Project (Walkowiak et al. 2020). The bread wheat cultivar Jagger was obtained from Wheat Genetics Resource Center (WGRC) at Kansas State University (accession number TA2909). The bread wheat cultivar CDC Stanley was obtained from the University of Saskatchewan. In addition to Jagger and CDC Stanley, whole-genome sequence data from another two wheat cultivars SY Mattis and Mace that potentially carry 2N^v^S segment were also utilized. Walkowiak et al. (2020) have further detailed about these plant materials. The diploid species *Ae. uniaristata* Vis. (2*n* = 2*x* = 14, NN, accession numbers TA2685 and TA2688), *Ae. tauschii* Coss. (2*n* = 2*x* = 14, DD, accession number TA2450) and *T. monococcum* L. (2*n* = 2*x* = 14, AA, accession number TA4342L96) were used for genomic in situ hybridization (GISH) analyses and were also obtained from the WGRC.

For 2N^v^S frequency and yield association analysis in the USA, two sources of wheat breeding lines were used: (1) Kansas State University wheat breeding program (1996–2017) composed of 6503 breeding lines (Supplemental Table S1). These lines were present in one or more breeding stages including F4/F5 individual plant short row (IPSR, 4721 lines, Supplemental Table S2), preliminary yield nursery (PYN, 1835 lines, Supplemental Table S3), advanced yield nursery (AYN, 403 lines, Supplemental Table S4) and Kansas interstate nursery (KIN, 122 lines, Supplemental Table S5). (2) USDA Regional Performance Nursery (USDA-RPN) testing program (1992–2015) of 825 lines from central US winter wheat breeding programs (Supplemental Table S6). These lines are from over 20 public and private breeding programs in the central USA including Texas A & M University, Oklahoma State University, Kansas State University, Colorado State University, University of Nebraska, South Dakota State University, North Dakota State University, Montana State University, Westbred, Bayer Crop Science and Limagrain (Rife et al. 2018, 2019).

For CIMMYT breeding programs (1996–2017), a total of 32,651 lines were used. These lines are from different yield nurseries including the (1) first-year yield trials (YTs) including 2014 YT (7,276 lines), 2015 YT (7,165 lines), 2016 YT (7,212 lines) and 2017 YT (8,752 lines), (2) second-year elite yield trials, EYTs: 2015 EYT (760 lines), 2016 EYT (838 lines) and 2017 EYT (828 lines), and (3) South Asia bread wheat yield trials (SABWYTs) from 2015 SABWYT (532 lines), 2016 SABWYT (525 lines), 2017 SABWYT (528 lines) and 2018 SABWYT (538 lines). The elite spring wheat yield trial lines (ESWYTs) from 1996 to 2017, which includes approximately 50 lines each year for a total of 1090 lines (Supplemental Table S7), were used to investigate the frequency changes for 2N^v^S over the corresponding years for CIMMYT programs.

### Cytogenetic characterization of 2N^v^S translocation in Jagger wheat

Preparations of mitotic and meiotic chromosomes followed protocols described in (Zhang et al. 2001; Koo et al. 2017) and all slides were stored at − 70 °C until use. The GISH-FISH procedure was according to a previously published protocol (Zhang et al. 2001; Koo et al. 2017). The probes were labeled with either digoxigenin-11-dUTP or biotin-16-dUTP according to the manufacturer’s instructions (Roche, Indianapolis, IN). Unlabeled total genomic wheat DNAs were used as a blocker. After post-hybridization washes, the GISH probes were detected with Alexafluor 488 streptavidin (Invitrogen, Grand Island, NY) for biotin-labeled probes and rhodamine-conjugated anti-digoxigenin (Roche) for digoxigenin-labeled probes. After recording the GISH signals, the slides were washed in PBS (10 mM sodium phosphate, 140 mM NaCl, pH 7.0) buffer three times (5 min each) and dehydrated in an ethanol series. The slides were reprobed with GAA repeats or pAS1 for FISH analysis. The FISH hybridization and post-hybridization washes were the same as for the GISH. Chromosomes were counterstained with 4′,6-diamidino-2-phenylindole (DAPI) in Vectashield anti-fade solution (Vector Laboratories, Burlingame, CA). Images were captured with a Zeiss Axioplan 2 microscope using a cooled CCD camera CoolSNAP HQ2 (Photometrics, Tucson, AZ) and AxioVision 4.8 software (Carl Zeiss Microscopy LLC, Thornwood, NY). The final contrast of the images was processed using Adobe Photoshop CS5 software. Chromosome measurements were done by ImageJ software (Schneider et al 2012).

### Genome sequencing, Hi-C anchoring of Jagger wheat and alignment of Jagger and Stanley version of 2N^v^S segments to reference Chinese Spring genome and to each other

Jagger DNA was extracted based on a modified CTAB method. Through the core facility at University of Illinois, paired-end (PE) and mate-pair (MP) libraries were made. We generated 14 lanes of Illumina HiSeq 2500 rapid run sequencing from 470 PE libraries, 8 lanes of Illumina HiSeq 2500 v4 sequencing for 800 bp PE libraries and 16 lanes of sequencing on Illumina HiSeq 4000 for 4 kb, 7 kb and 10 kb MP libraries yielding a total of 19 billion reads. The sequencing data were delivered to NRGene (Ness-Ziona, Israel) in Nov 2016 for assembly using DeNovoMAGIC^TM^3.0 assembly pipeline by incorporating 10X Chromium linked-read information (10X Genomics, Pleasanton, CA, USA).

The NRGene scaffolds were ordered and oriented based on chromosome contact frequencies using a custom R implementation (Beier et al. 2017) of the algorithm described in Burton et al. (2013). Chromosome conformation capture (Hi-C) sequencing libraries were generated for Jagger wheat using a plant-adapted tethered chromosome conformation capture (TCC) protocol (Kalhor et al. 2011; Himmelbach et al. 2018). Two independent assembly versions of the chromosome 2A (from Jagger and CDC Stanley) were constructed and aligned to the Chinese Spring reference genome using Burrows–Wheeler alignment (BWA-MEM, v0.7.17) tool (Li and Durbin 2009) with assembly derived overlapping reads of 5kb, step 1kb, as query, and Minimap2 (v2.5) (-I 5G -x asm5) (Li 2018) to delineate the *Ae. ventricosa* 2N^v^S segment. The two assemblies of 2N^v^S segment were also aligned to each other using BWA and Minimap2. When there was a disagreement in the order of scaffolds between Jagger and CDC Stanley, the scaffold breakpoints were manually checked, and a consensus order and orientation was constructed based on mis-orientation at the scaffolds break points. Minimap2 (Li 2018) (paftools.js call) was used to call variants between the 2N^v^S segment of the different varieties (Jagger and CDC Stanley). To verify the identified polymorphisms, we also mapped 17 × raw reads of CDC Stanley (470 PE) and Jagger to reference Jagger genome using BWA and called SNPs using BCFtools (mpileup -q 20 -a ‘DP,DV,’ call -mv -f GQ) and filtered SNPs using an awk tool: http://bitbucket.org/ipk_dg_public/vcf_filtering. To delineate the extent of the original VPM1 translocation, which would include 2N^v^S *ventricosa* translocation segment and longer wheat chromatin segment inherited from VPM1 wheat 2A chromosome, we aligned raw reads (15X–20X) of 2N^v^S carriers SY Mattis and Mace to the Jagger reference genome using BWA and called SNPs using BCFtools and above awk tool with minDP 40 and otherwise default parameters. Only SNPs overlapping between assembly to assembly calls (Minimap2 paftools.js call) and raw reads alignment based SNP calling (BWA+BCFTOOLS+AWK) were reported.

### RNA collection and sequencing, gene model prediction

For transcript profiling, Jagger plants were grown in an environmentally controlled, 15 square foot growth chamber at the University of Minnesota in 2016. The growth and vernalization treatment conditions and tissue types sampled are detailed in Supplemental Table S8. A total of 15 different tissue types throughout the full growth cycle were collected in biological triplicate by flash freezing in liquid nitrogen. RNA was extracted using the Qiagen Plant RNeasy kit. RNA samples were used to generate Illumina libraries that were sequenced on the Illumina HiSeq2500 platform at the DOE Joint Genome Institute.

Structural gene predictions of *T. aestivum* Jagger chromosome 2A were done by merging ab initio prediction and information obtained by mapping of de novo assembled transcriptome data. First, we mapped the RNA-seq datasets against the reference genome sequence of Jagger using STAR (STAR_2.6.0b) (Dobin et al. 2013). Based on these alignments intron and exon structures were generated, which were subsequently fed into ab initio prediction by AUGSTUS (version 3.2.3) (Stanke et al. 2006) using the default wheat model. Second, we de novo assembled all RNA-seq datasets into transcripts (Trinity 2.5) (Grabherr et al. 2011) and mapped the assembled transcripts back to the genome using GMAP (version 2017–06-20) (Wu and Watanabe 2005). EvidenceModeler (version 1.1.1) (Haas et al. 2008) was used to combine the ab initio predictions and mapped transcripts, resulting in a set of > 60 K candidate gene models.

To differentiate candidates derived from EvidenceModeler into complete and *bona-fide* genes, non-coding transcripts, pseudogenes and transposable elements, we further applied a confidence classification protocol similar to that previously applied for Chinese Spring (IWGSC 2018). Candidate protein sequences were compared against the following 3 manually curated databases: (1) **PTREP**, (2) **UniPoa** and (3) **UniMag,** to classify the genes into high-confidence (**HC**) genes and low-confidence (**LC**) genes. Finally, we assigned a functional annotation including human readable description lines to the gene models that were classified as HC using the Automated Assignment of Human Readable Descriptions (AHRD) pipeline (version 1.6; http://github.com/groupschoof/AHRD). Representative coding sequences for **HC** 2N^v^S genes and 2AS, 2BS, 2DS genes from Chinese Spring (IWGSC 2018) were aligned to each other using BLAST. Reciprocal best blast hits from the alignments were visualized using Circos (Krzywinski et al. 2009).

### Phylogenetics of NLR genes and NLR gene transcription analysis

A total of 58 AHRD annotated nucleotide-binding leucine-rich repeat (NLR) genes from Jagger 2N^v^S region and 65 AHRD NLR genes from the corresponding Chinese Spring 2A segment (IWGSC 2018) were combined and their phylogenetic relationships were analyzed using MEGA 7 software (Kumar et al. 2016), with the maximum likelihood method. The tree was visualized using ggtree package of R (Yu et al. 2017).

Raw RNA-seq reads (~ 3 billion) were aligned to refseq v1 gene models (IWGSC 2018) together with newly assembled 2N^v^S gene models using Kallisto tool (Bray et al. 2016) to provide transcript abundance estimates on a gene level. RNA-seq read counts were normalized using edgeR (Robinson et al. 2009) and limma (Smyth 2005). Transcript levels for NLR genes were hierarchically clustered and visualized using heatmap.2 function in R.

### Predicting the presence of 2N^v^S segment in various breeding lines based on GBS and reference alien and wheat genomes

Genotyping by sequencing (GBS) (Poland et al. 2012) was conducted for breeding lines in KS, USDA-RPN and CIMMYT programs. The GBS key files listing plant names, sequencing flowcell IDs, lanes, barcodes, etc. are given in Supplemental Tables S9-S11. Predicting the presence or absence of alien 2N^v^S segment was conducted in two steps. (1) The discovery step aims to identify alien 2N^v^S or common wheat-specific GBS tags. Briefly, a training set of 2N^v^S positive and negative breeding lines was identified based on marker data (Helguera et al. 2003). TASSEL (Bradbury et al. 2007) GBSv2 pipeline was used to extract ‘tag by taxa (TBT)’ file after alignment to alien and wheat reference genomes. Tags that show > 99% of distribution in alien positive lines are deemed alien 2N^v^S-specific, whereas tags that show > 99% distribution in alien negative lines are deemed common wheat 2AS-specific. (2) The production step aims to predict the presence or absence of alien 2N^v^S segment for large set of breeding lines. Again, GBS reads were processed using the TASSEL GBSv2 pipeline. To avoid the excessive use of computing resources and storage capacities, we developed/modified a TASSEL plug-in to export only ‘selected tag by taxa’ files (github.com/umngao/Tassel5TagTaxaSel). The functionality of this TASSEL plugin was further incorporated into an official TASSEL build v5.2.66 (Oct 29, 2020), with minor modifications by the Cornell TASSEL admin team. After extracting selected TBT file, an R function was created to produce the alien 2N^v^S prediction based on inputs of i) alien-specific tags, ii) wheat-specific tags, iii) selected TBT files and iv) raw reads count per sample (http://github.com/umngao/alien_2ns_predict). The accuracy of prediction was validated using a wet lab assay (Ventriup-Ln2) (Helguera et al. 2003).

### Statistical association of 2N^v^S with grain yield and various other traits

For the KS breeding program, phenotype data were classified into different breeding stages IPSR, PYN, AYN and KIN. For IPSR and PYN studies, the experiments were conducted using modified augmented design method (Type 2) with a Method 1 adjustment according to Lin and Poushinsky (1985). For AYN and KIN, the experiments were conducted using an Alpha (0,1) design (generalized lattice) according to Patterson and Williams (1976). The data were analyzed using AGROBASE software (Agronomix Software Inc, Winnipeg, Manitoba, Canada) to accommodate different design aspects of the experiments. Then the best linear unbiased prediction (BLUP) values for each genotype were retrieved on a per year and location basis (Supplemental Table S12). These individual values were used in mixed model analysis to estimated 2N^v^S effects, with the presence or absence of 2N^v^S as having fixed effects and locations as having random effects implemented in the R package lmerTest. We studied the association of 2N^v^S with yield under different KS wheat breeding stages by dividing the data into stage-specific datasets.

We also obtained yield data for 825 wheat breeding lines over the past two decades (1992–2015) through the USDA-RPN website (Supplemental Table S13) (Rife et al. 2018). The RPN genotype data analyzed in this study were generated by Rife et al (2018). For fitting of linear mixed models, 2N^v^S was treated as having fixed effects, sites (and years when analyzing all years data together) as having random effects implemented in the R package lmerTest. To further verify the effects of 2N^v^S on yield, we also ran genome wide association study using TASSEL for USDA-RPN dataset.

For the CIMMYT breeding program, the YTs (2 replications), EYTs (3 replications) and ESWYTs (3 replications) were planted at the Norman E. Borlaug Research station, Ciudad Obregon, Sonora, Mexico, in several trials, with each trial comprising 28 lines and two checks in an alpha-lattice design. The trials were sown in mid-November in optimally irrigated environments that received 500 mm of water under the bed planting system. Similarly, the SABWYTs (3 replications) were planted at several locations in South Asia including Ludhiana, Jabalpur and Pusa in India, Faisalabad in Pakistan and Jamalpur in Bangladesh. For all the nurseries, the harvested grain weight calculated on a plot basis was used as a measure of grain yield (Supplemental Table S14–S17). The best linear unbiased estimates (BLUEs) for grain yield were calculated using the ASREML statistical package (Gilmour 1997), via the following mixed linear model:

$$y_{ijkl} = \mu + g_{i} + t_{j} + r_{k(j)} + b_{l(jk)} + \varepsilon_{ijkl}$$

where ${y}_{ijkl}$ is the observed GY, μ is the overall mean, ${g}_{i}$ is the fixed effect of the genotype, ${t}_{j}$ is the random effect of the trial that is independent and identically distributed (IID) (${t}_{j}\sim N (0, {\sigma }_{t}^{2})$), ${r}_{k(j)}$ is the random effect of the replicate within the trial with IID ${(r}_{k(j)}\sim N (0, {\sigma }_{r}^{2}))$, ${b}_{l(jk)}$ is the random effect of the incomplete block within the trial and the replicate with IID (${b}_{m(jk)}\sim N (0, {\sigma }_{b}^{2}))$ and ${\varepsilon }_{ijkl}$ is the residual with IID ${(\varepsilon }_{ijkl}\sim N (0, {\sigma }_{\varepsilon }^{2}))$.

The YTs (YT 2013 and YT 2014) were also evaluated for lodging on an ordinal scale from zero to five, whereas the EYTs were evaluated for rust disease resistances. We then performed tests of association of grain yield, lodging and disease resistance with the 2N^v^S segment in all the nurseries using a simple linear regression model with grain yield BLUEs as the response variables and the presence or absence of the 2N^v^S segment as fixed effect predictors.

## Results

### Cytogenetic evidence of Aegilops ventricosa 2N^v^S translocation in wheat

**Fig. 1:**
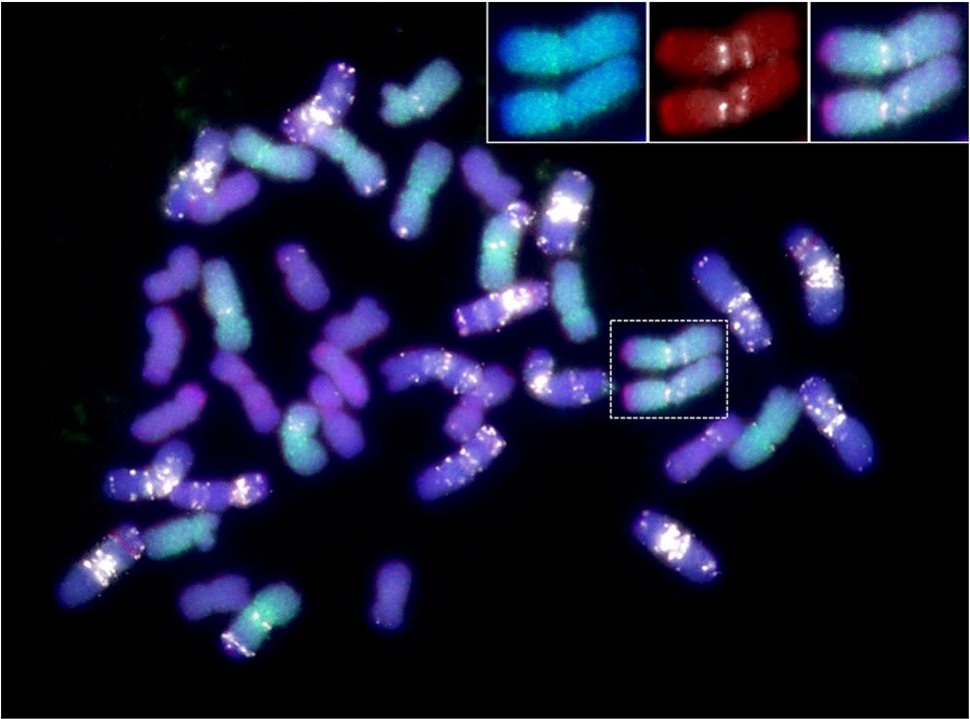
Sequential GISH-FISH patterns of mitotic chromosomes of Jagger, labeled with genomic *Ae. uniaristata* (N-genome, visualized in red), *T. urartu* DNA (A-genome, visualized in green) and GAA repeats (visualized in white). Inserts: enlarged images showing the 2N^v^S-2AS translocation. Inserts show GISH-FISH patterns of chromosome 2A

**Fig. 2:**
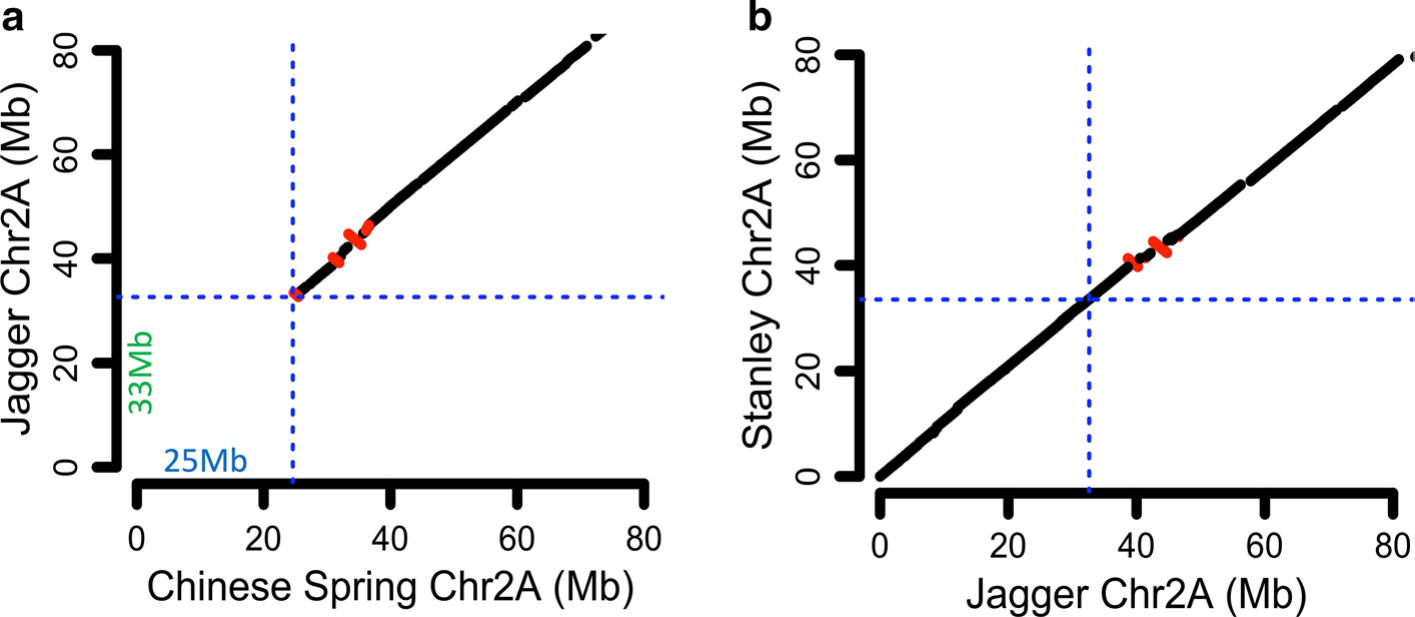
Physical delineation of 2N^v^S segment **a** Comparative dot plot alignment of Jagger chromosome 2A and Chinese Spring chromosome 2A (showing the first 80 Mb). **b** Jagger versus Stanley 2N^v^S region. Blue horizontal and vertical lines indicate the boundaries of 2N^v^S segment in Jagger and its corresponding section in Chinese Spring or Stanley. Segments in red indicate reverse alignment orientation

We utilized total genomic DNA from *Ae. uniaristata* Vis. (2*n* = 2*x* = 14, NN) as a probe to detect the presence of 2N^v^S segment in Jagger wheat. We found hybridization signals at the distal end of the short arm of wheat chromosome 2A (Fig. 1 and supplemental Figure S1), in agreement with the results of comparative dot plot analysis (Fig. 2a) and the previously known position of the original VPM1 translocation. Cytological measurements (*n* = 5) revealed that the translocation segment comprised roughly 6.5% of chromosome 2A of Jagger. Though not a target of this study, chromosome painting using A-genome and D-genome DNA as probes also revealed the presence of an intergenomic translocation in Jagger on 2DL from an unidentified A-genome chromosome (Supplemental Figure S2).

### Physical assembly and delineation of the 33 Mb 2N^v^S segment

We sequenced the Jagger and CDC Stanley wheat genomes using a combination of Illumina short read technology and 10X Genomics linked reads (Weisenfeld et al. 2017). Using the NRGene DenovoMagic 3.0 pipeline, the Jagger and CDC Stanley reads were assembled into 141,382 and 105,606 scaffolds, totaling 14.54 and 14.46 Gb, respectively. To construct 21 pseudomolecules for Jagger and CDC Stanley, we used high-density genetic maps (Chapman et al. 2015) and chromosome conformation capture data (Lieberman-Aiden et al. 2009; Mascher et al. 2017) and anchored 3116 and 1592 scaffolds, respectively. The anchored and oriented scaffolds total 14.17 and 14.21 Gb, representing > 97% of the total assemblies for both genomes. The other 10 + Wheat Genomes including those of SY Mattis and Mace had similar assembly completeness (Walkowiak et al. 2020).

**Fig. 3:**
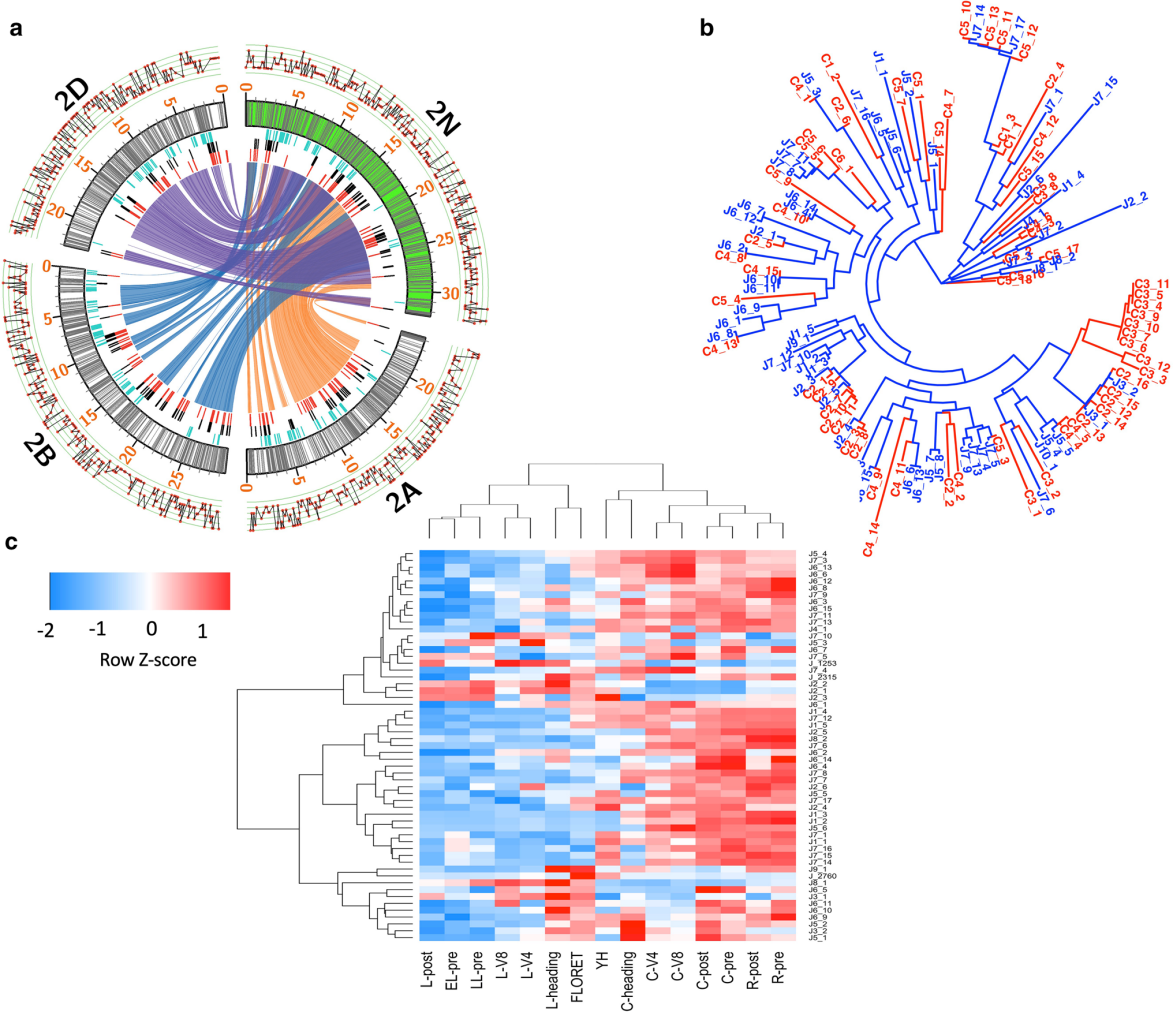
Gene contents, NLR phylogeny and resistance gene transcription patterns. **a** Conserved gene orders for 2 N genes compared to 2A, 2B, 2D genes. Outer layer line plot track indicates gene density (count 1–5) per 100 kb region; labeled track are chromosomes with 2N^v^S filled with green color; inner tracks show positions of AHRD annotated cytochrome P450 (light blue) and NLR genes based on NLR-Annotator (black) and AHRD annotation (red). Links connecting orthologous genes between 2 N (green) and common wheat chromosomes (no fill color). **b** Phylogenetic relationships of 2N^v^S NLR genes compared to Chinese Spring 2AS NLRs. C (red) denotes Chinese Spring, and J (blue) denotes Jagger genes. **c** Hierarchical clustering of resistance gene expression patterns. Tissue types are: early leaf (EL); leaf (L); late leaf (LL); root (R); crown (C); young head (YH); and single floret from a young head (FLORET). Tissue growth stages are: pre-vernalization (pre); 4th week of vernalization (V4); 8th week of vernalization (V8); 1 week post-vernalization (post); and heading (heading)

The 2N^v^S region was delineated based on comparative dot plot alignments of Jagger chromosome 2A to the previously sequenced Chinese Spring chromosome 2A (IWGSC 2018) and CDC Stanley chromosome 2A (Fig. 2a). In this alignment, lower-quality alignments such as those with percent identity below 40% were not included, as they might be suggesting cross-genome (2 N-2A) alignments (see Fig. 3a for details). The dot plot displayed much noise (but still showed the introgression segment) if including all alignments, regardless of percent identity (Supplemental Figure S3). The size of the 2N^v^S segment was 32.53 Mb and 33.49 Mb in the Jagger and CDC Stanley assemblies, respectively, compared to a smaller 24.64 segment in the native wheat 2A chromosome of Chinese Spring. There were 21 scaffolds for Jagger and 11 scaffolds for CDC Stanley for the 2N^v^S region (Supplemental Table S18). We used a comparative approach to correct scaffold ordering and orientations in Jagger and CDC Stanley 2N^v^S segment by manual alignment of individual breakpoints of scaffolds, finding the disagreement in scaffold orientation between Jagger and CDC Stanley and correcting the genome assembly where the change in orientation corresponded to scaffold endpoints. The true order of scaffolds could therefore be established since the two versions of assembly did not break at the same positions. The total assembled sizes for chromosome 2A in Jagger and CDC Stanley are 804 and 803 Mb, respectively; thus, the 2N^v^S segment occupies roughly 4% of the chromosome, comparable to the cytological estimate.

We also explored polymorphisms between Jagger and CDC Stanley versions of 2N^v^S segments and found relatively low number of variants (23 SNPs for the 33 Mb region) (Supplemental Table S19). These variants likely reflect accumulated mutations in the recent breeding history (~ 10–20 generations) from a common original segment introgression. The larger (by 0.9 Mb) size of the CDC Stanley 2N^v^S assembly is partially explained by the larger total gap size in CDC Stanley (1.36 vs. 0.85 Mb). The order and orientations among the two assemblies are highly consistent after manual correction of orders based on breakpoints alignment (Fig. 2b). We aligned markers from a previous study (Xue et al. 2018) and found that all 2N^v^S-specific markers aligned to the 2N^v^S region (0–32.6 Mb) of Jagger chromosome 2A, and the adjacent monomophic markers following 2N^v^S aligned to (33-39 Mb) of Jagger chromosome 2A (Supplemental Table S20). These results support the observed delineation of the 2N^v^S segment in Jagger in this study. Our results also show that there is a 3 to 6 Mb (position 33-39 Mb on chr2A) region following the *Ae. ventricosa* segment that is low in polymorphisms among the four 2N^v^S carriers (Jagger, CDC Stanley, SY Mattis and Mace) and expected to be wheat chromatin with identity by descent (IBD) from the original VPM1 translocation (Supplemental Figure S4).

### Gene content of 2N^v^S segment, NLR phylogeny and resistance gene transcription patterns

To examine gene content of 2N^v^S, we annotated Jagger chromosome 2A using a combination of ab initio prediction and RNA-seq evidences. A total of 7593 genes were annotated for Jagger chromosome 2A (Supplemental Table S21). A total of 535 high-confidence genes within the 2N^v^S segment were annotated. To examine macro-collinearity, we then compared the 2N^v^S segment to the corresponding region on chromosomes 2A, 2B and 2D of hexaploid wheat (Fig. 3a, Supplemental Table S22). We found the overall gene order to be highly conserved between the N-genome segment and the three wheat genomes.

Given the known importance of 2N^v^S disease resistance genes, we closely examined the nucleotide-binding domain leucine-rich repeat (NLR) containing genes of this segment. From the functional annotations, we found 58 NLR disease resistance genes, accounting for > 10% of the total number of genes (535) annotated for the region (Supplemental Table S21). The percentage of NLRs is similar to that in Chinese Spring (65/588 = 11%) (IWGSC 2018). However, the annotation procedures used in this study are not the same as previously used Chinese Spring. To make a more direct comparison, we ran ab initio annotation of NLR genes for both Jagger and Chinese Spring using NLR-Annotator (Steuernagel et al. 2020) and found that the predicted number of NLR genes for 2N^v^S (*n* = 58) is much higher compared to the same segment in the Chinese Spring reference genome (*n* = 35) (Fig. 3a, Supplemental Table S23, Supplemental Figure S5). We also detected a few ABC transporter genes in this region (Supplemental Table S21). These could potentially be candidates for disease resistance, as demonstrated in the case of *Lr34*, where an ABC transporter provided effective resistance against multiple fungal pathogens (Krattinger et al. 2009). In addition, there is an abundance of > 10% cytochrome P450 genes potentially involved in plant development and resistance to stresses (Fig. 3a, Supplemental Table S21).

To test the hypothesis that 2N^v^S might carry unique NLR gene families that are partially responsible for the suite of disease resistance genes found on this segment, we also examined the phylogenetic relationships of 2N^v^S NLR genes compared to those present in the homeologous segment of Chinese Spring chromosome 2A. This comparison used 58 NLRs from Jagger and 65 from Chinese Spring for the phylogenetic analysis. To help visual representation, gene IDs were annotated based on physical position, i.e., genes starting with the same numeric letters are physically closer on the genome (Supplemental Table S24). We identified potential sub-clades of NLRs that appear to be unique to chromosome 2N^v^S such as J7_7, J7_8 and J7_11, or chromosome 2A, such as C3_4, C3_5 and C3_6 (Fig. 3b).

To further understand what genes from this large suite of resistance genes might be important in conferring known disease resistance phenotypes, we examined the transcription patterns of disease resistance genes for the 2N^v^S region (Fig. 3c). Under the hypothesis that the 2N^v^S segment carries genes for a large diversity of pathogens that impact different parts of the plant, we would expect gene transcripts that are tissue-specific and could be prioritized as candidates depending on the specific pathogen lifestyle. We found groups of NLR genes that are differentially transcribed across tissues (Fig. 3c). It can be hypothesized for example that the NLR with increased transcript accumulation in head tissues, such as J_2760 (ABC transporter) and J2_3, may be involved in wheat blast resistance, whereas those that exhibit increased transcript accumulation in root tissues, such as genes J8_2, J1_2 and J1_3, may be involved in nematode resistance. A few genes with increased transcript accumulations in the leaves such as genes J2_1, J2_2 may be involved in resistance to leaf or stripe rust diseases. Further investigation is underway to pinpoint exactly which genes confer resistance to the different pests and diseases.

### 2N^v^S frequency in wheat breeding programs and impact on grain yield

**Fig. 4:**
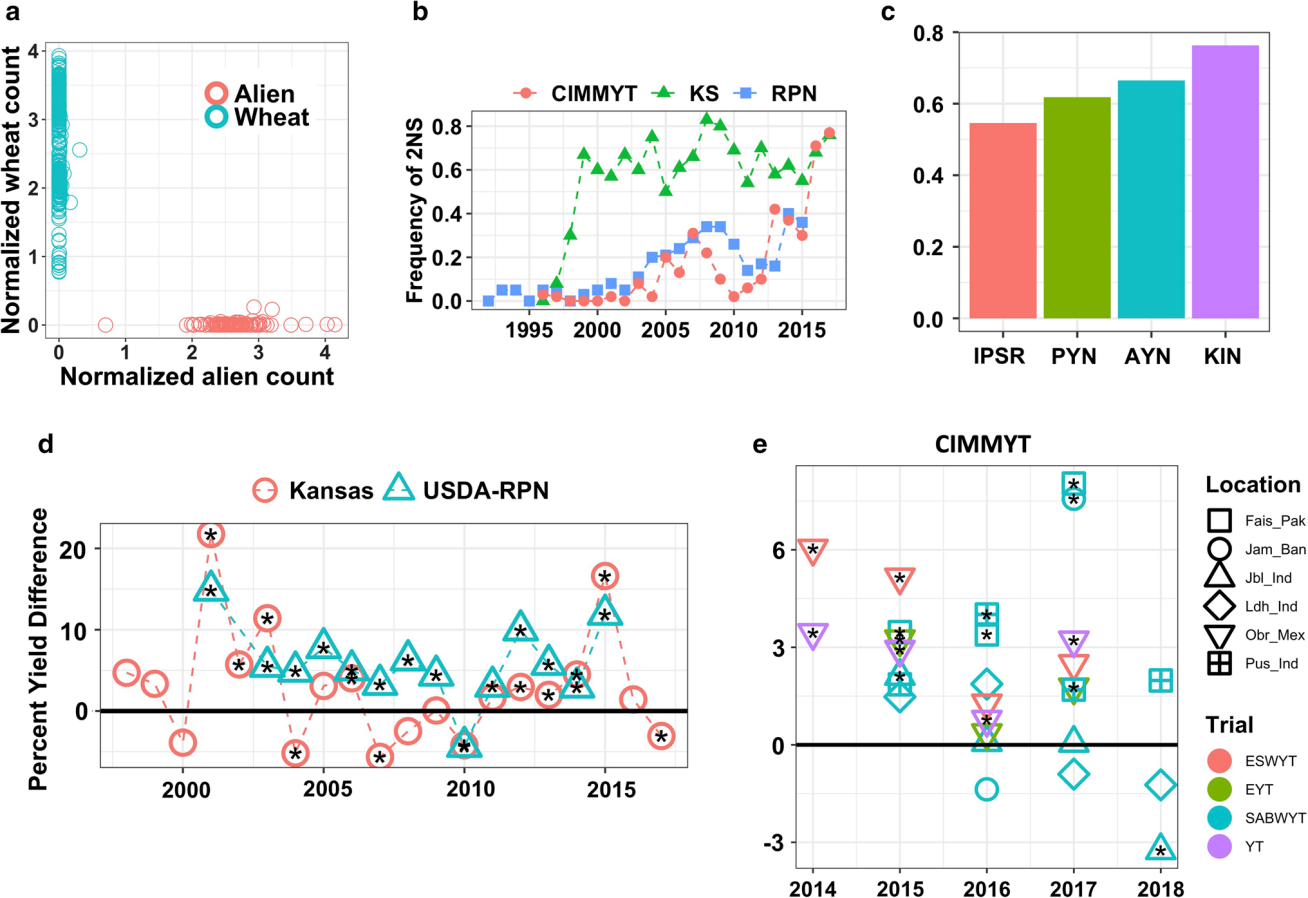
Predicting 2N^v^S presence based on GBS data and 2N^v^S impact on breeding. **a** 2N^v^S positive and negative breeding lines based on relative counts of alien or wheat GBS tags. The prediction of 2N^v^S presence for over 40 thousand breeding lines in various programs show for 2N^v^S positive (red) and negative (blue). **b** 2N^v^S frequency by year for US winter wheat (KS and USDA-RPN) and CIMMYT spring bread wheat breeding program. **c** 2N^v^S frequency over major KS wheat breeding stages showing first year to final year yield testing in order (*IPSR* Individual Plant Short Row; *PYN* Preliminary Yield Nursery; *AYN* Advanced Yield Nursery; *KIN* Kansas Interstate Nursery). **d** Estimated yield effect difference of 2N^v^S positive vs negative lines in US winter wheat breeding program. **e** Estimated effect of 2N^v^S on CIMMYT wheat yield showing percentage yield difference between 2N^v^S and non-2N^v^S lines.* X*-axis: years of evaluation, Y-axis: percent yield difference, positive values indicate that the 2N^v^S effect is providing a yield effect. Shapes with black asterisks represent environments with statistically significant yield differences between 2N^v^S and non-2N^v^S lines. ESWYT, elite spring wheat yield trial lines; EYT, elite yield trials; SABWYT, South Asia bread wheat yield trials; YT, yield trials. Fais_Pak, Faisalabad in Pakistan; Jam_Ban, Jamalpur in Bangladesh; Jbl_Ind, Jabalpur in India; Ldh_Ind Ludhiana in India; Obr_Mex, Obregon Mexico; Pus_Ind, Pusa in India

Finally, we tested the hypothesis that 2N^v^S has a yield benefit (or corresponding yield penalty) depending on the presence or absence of pathogens to which 2N^v^S carries effective resistance genes. To examine the effect of 2N^v^S across a broad range of breeding lines, we built a bioinformatics pipeline using genotyping -by -sequencing (GBS) data to predict the presence or absence of the 2N^v^S segment. The prediction was based on relative counts of wheat or alien chromatin-specific tags (Fig. 4a). The method was validated by screening 135 doubled haploids lines with marker Ventriup-LN2 (Helguera et al. 2003) and proved to be highly accurate (> 99%) (Supplemental Table S25). The method is useful for predicting the presence or absence of 2N^v^S based on GBS data, while removing the labor of further wet lab verifications.

We then studied the 2N^v^S carrier frequency changes over the past two and a half decades for Kansas winter wheat, central USA regional winter wheat programs (USDA Regional Performance Nursery) and the CIMMYT spring wheat breeding program. Over each of the programs, we found a large increase in the 2N^v^S frequency from the early 90 s through to present (Fig. 4b). This supports a hypothesis that 2N^v^S is indeed a beneficial allele in the wheat varieties and positive selection pressure is driving an increase in the allele frequency. We observed a drop in 2N^v^S frequency around the year 2008–2010, likely reflecting the loss of *Yr17* resistance against wheat yellow rust disease due to changes in pathogen virulence. It is noteworthy that the 2N^v^S frequency in recent years for both KS and CIMMYT breeding programs approach 80%, indicating strong selection in the programs. To further examine the selection of 2N^v^S in the breeding programs, we examined the frequency of 2N^v^S across advancing stages of selection. The increase in 2N^v^S frequency is also consistent when examining yield testing stages in the KS program (Fig. 4c). As the breeding lines are selected and advanced in the breeding pipeline, the corresponding 2N^v^S frequency is increasing.

We ran a mixed model analysis to account for 2N^v^S presence, year, test nursery and breeding program to determine the impact on yield of the 2N^v^S segment on yield per se (Fig. 4d, Supplemental Table S26). We found that the presence of 2N^v^S in central US winter wheat breeding lines had a positive effect on grain yield over the period studied. Seventeen out of 19 years when there was a significant difference, the 2N^v^S segment was the favorable allele with positive effect on yield. The positive effect remained significant across breeding programs (KS or USDA-RPN) and breeding stages and remained significant after correcting for kinship effects (Supplemental Figure S6). For CIMMYT programs, we observed a similar trend, there is a consistent yield advantage for 2N^v^S lines (Fig. 4e). For example, under optimal irrigation conditions, the presence of 2N^v^S segment is giving a yield boost of approximately 1.7% (0.09–0.12 tons/ha). For yield, a low heritability trait with complex genetic architecture, this is a substantial effect for a single locus. In addition to yield, our study also suggests that 2N^v^S is positively associated with resistance to crop lodging (Supplemental Table S27).

## Discussion

There is a rich history in wheat breeding of impactful translocations coming from distant species and wild relatives. For example, the wheat–rye 1RS translocations in wheat is frequently associated with higher yield and stress tolerance and was brought into hundreds of wheat cultivars worldwide (Lukaszewski 2015; Graybosch et al. 2019; Rabanus-Wallace et al. 2019). The T7DL·7Ag translocation in wheat from *Thinopyrum elongatum* (syn. *Agropyron elongatum* or *Lophopyrum elong*atum) is associated with rust resistance and yield benefits (Singh et al. 1998; Monneveux et al. 2003; Ceoloni et al. 2014). The wheat *Fhb7* gene was a recent and excellent example of horizontal gene transfer of a fungus gene into wheat via wild relative (Wang et al. 2020). These novel introgressions have provided new genetic diversity that provides disease and insect resistance as well as stress tolerance. Here, we have shown this to be the case with the 2N^v^S translocation from *Ae. ventricosa*. Across a survey of multiple breeding programs, we find the translocation at very high frequency, increasing over the years and across major breeding stages, indicative of the importance and yield impact of the 2N^v^S in the USA and around the globe.

This is the first report in hexaploid wheat visualizing the 2N^v^S segment translocated to chromosome 2A using GISH analysis. The 2N^v^S fragment was previously estimated to be around 25–38 cM in genetic distance based on 2A^m^S corresponding genetic map information (Helguera et al. 2003); however, no physical or cytological estimate of the size was made. We report the first assembly of the 2N^v^S segment and estimate the physical size at 32.6–33.5 Mb. This size differs from an estimate of 27.8 Mb based on genetic mapping and positioning on the wild emmer wheat reference genome (Avni et al. 2017; Xue et al. 2018), which is closer to the smaller physical size of the 2A segment that was replaced. This estimated segment size based on homology hits on reference genomes, underestimated the size by 20% relative to the de novo genome assembly presented here. The 2N^v^S segment is larger than most of the individual rice chromosomes and increased the size of chromosome 2A by approximately 9 Mb. Given that the segment is located at a gene-rich terminal section of the chromosome, which is well assembled, we feel confident to postulate that the actual size of this 2N^v^S segment is roughly 33 Mb.

The 2N^v^S translocation was originally transferred and selected for eyespot disease resistance and subsequently found to confer resistance to multiple diverse diseases (Bariana and McIntosh 1994; Williamson et al. 2013; Cruz and Valent 2017). In line with this, we found that the 2N^v^S segment is indeed rich (> 10%) with NLR-type genes, as would be postulated for a telomeric region carrying resistance to many different pathogens. We found an increased number of NLRs relative to the replaced wheat segment in the Chinese Spring reference genome and observed differential expression of the NLR genes across tissues, supporting the hypothesis that different genes, or sets of genes, are conditioning resistance to the diverse array of pathogens and pathogen lifestyles (Bariana and McIntosh 1994; Jahier et al. 2001; Williamson et al. 2013; Cruz et al. 2016). This assembled sequence of 2N^v^S will pave the way for accelerated efforts to identify and clone resistance genes residing on this translocation. The assembly of 2N^v^S is particularly useful for identifying these resistance genes as the translocation is non-recombining with wheat, which makes the alien genes recalcitrant to map-based cloning. Guided with a high-quality reference, mutation-based mapping of genes on the segment is now possible.

We developed a bioinformatics pipeline to predict alien 2N^v^S presence based on historically available GBS datasets. The new pipeline is likely to be adapted to genotype other wheat alien introgressions. For instance, our group applied similar steps in predicting wheat streak mosaic virus (*Wsm1*) (Lay et al. 1971) and barley yellow dwarf (*bdv2*) (Hohmann et al. 1996) resistance based on GBS data (manuscript in prep). The current availability of extensive GBS or other skim sequencing data in various breeding programs in the world will likely benefit from this pipeline that allows SNP calling as well as simultaneous genotyping of multiple important agronomic traits.

We initially hypothesized that breeder selection of 2N^v^S would be driven by disease resistance. However, we find evidences that 2N^v^S is giving yield benefits per se, even in the absence of disease pressure. First, the CIMMYT breeding location of Cd. Obregon in Mexico is virtually disease free and the 2N^v^S segment is giving a consistent yield advantage in this environment (Fig. 4e). Second, we did not observe correspondence between the 2N^v^S yield advantage (or infrequent yield reduction) in the KSU and RPN datasets and any of the years with significant disease pressure, e.g., years 2007, 2010, 2012, 2015, 2016 and 2017 (Hollandbeck et al. 2018). Finally, *Yr17-*derived resistance on the 2N^v^S segment was defeated by virulent race(s) in 2008. However, we did not observe a corresponding decrease in allele frequency in the KSU breeding materials following 2008 as would be expected if the segment had a detrimental effect on yield and was not providing any effective disease resistance. Overall, these observations support a hypothesis that the 2N^v^S is actually conferring yield advantage, even in the absence of pathogen pressure.

While further insight is needed, there appear to be certain physiological and yield benefits to the 2N^v^S segment that are independent of the disease resistance. For example, this and our previous studies suggest that 2N^v^S is associated with crop lodging resistance. The exact physiological mechanism regarding how the 2N^v^S segment provided positive crop yield benefits remains to be elucidated. Regardless, the fact that the frequency of this segment is present at high frequency in many breeding programs and found in germplasm around the world supports its overall value in wheat improvement. The observation that 2N^v^S is increasing over major breeding stages when selection was primarily done by yield shows the positive breeding value of this segment whether indirectly from disease resistance or some agronomic benefit or yield increase per se. Our results suggest that the 2N^v^S segment was selected in the yield testing by harvesting combines in the field, and the yield effect is prominent over breeding stages and across a wide range of wheat germplasm and target environments.

The high frequency of 2N^v^S across multiple winter and spring wheat breeding programs supports that this important translocation is being readily selected by breeders, perhaps from partial or residual disease resistance from the multitude of resistance genes on 2N^v^S or directly from favorable yield effects independently from any effective resistance. The release of varieties carrying 2N^v^S from these various programs shows that this translocation is collectively grown on up to hundreds of millions of hectares throughout the world and is having a large impact on wheat productivity. Detailed characterization of alien translocation segments such as 2N^v^S in the post-genomics era of wheat will give important insight into the wheat pan-genome and will have significant implications for wheat breeding and improvement.

## Electronic supplementary material

Below is the link to the electronic supplementary material.

Supplementary file1 (XLSX 13614 kb)

Supplementary file2 (PDF 5316 kb)

## Data Availability

The raw sequencing reads for Jagger and Stanley can be accessed via NCBI SRA PRJNA544491. The de novo assembled genomes for Jagger and CDC Stanley can be downloaded at https://wheat.ipk-gatersleben.de/. The RNA-seq data for Jagger were submitted to NCBI SRA with 45 accession numbers listed in supplemental table S8. The GBS data for Kansas, USDA-RPN and CIMMYT breeding lines can be accessed via PRJNA623575, PRJNA474575 and PRJNA498085. The 2N^v^S complete assembly, annotation, CDS and protein sequences are available at a DOI link https://dx.doi.org/10.5447/ipk/2020/21.

## Abbreviations

AHRD: Automated assignment of human readable descriptions
AYN: Advanced yield nursery
BLAST: Basic local alignment search tool
BLUP: Best linear unbiased prediction
BLUE: Best linear unbiased estimate
CDS: Coding sequence
CIMMYT: International Maize and Wheat Improvement Center
ESWYT: Elite spring wheat yield trial
EYT: Elite yield trials
GBS: Genotyping by sequencing
GISH: Genomic in situ hybridization
FISH: Fluorescence in situ hybridization
Hi-C: Chromosome conformation capture
IPSR: Individual plant short row
KS: Kansas
KIN: Kansas interstate nursery
NLR: Nucleotide-binding leucine-rich repeat
PYN: Preliminary yield nursery
RBH: Reciprocal best blast hit
SABWYT: South Asia bread wheat yield trials
USDA-RPN: United State Department of Agriculture Regional Performance Nursery
YT: Yield trials
