# Supplementary material for: The Aegilops ventricosa 2NvS segment in bread wheat: cytology, genomics and breeding
Source: Theor Appl Genet. 2020 Nov 12;134(2):529–42. doi: 10.1007/s00122-020-03712-y (PMC7843486; doi:10.1007/s00122-020-03712-y)
Supplement: Supplementary file 2 — Supplementary file2 (PDF 5316 kb) [file 122_2020_3712_MOESM2_ESM.pdf]

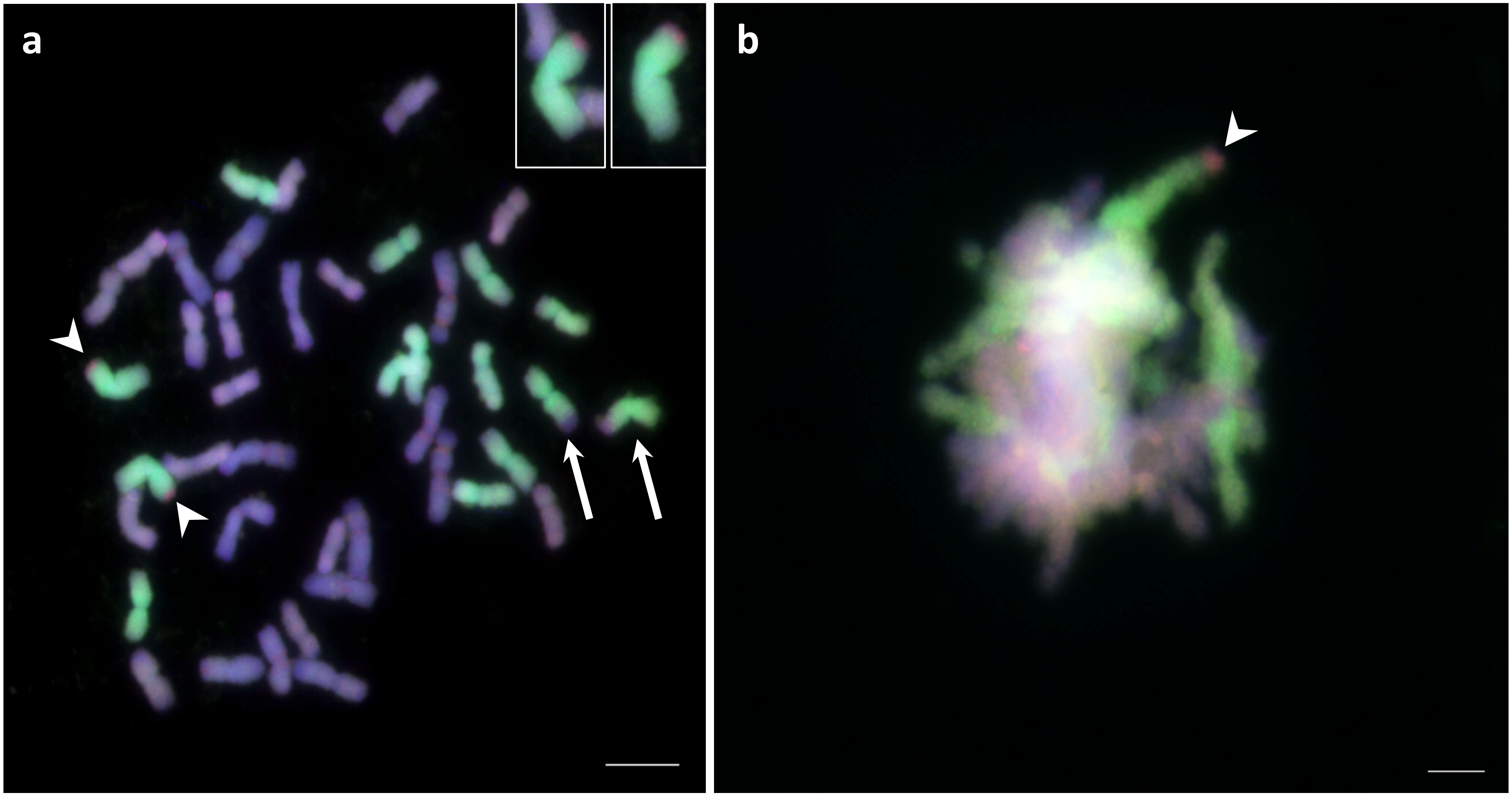

Supplemental Figure S1. GISH patterns of mitotic (a) and meiotic pachytene (b) cells of Jagger, labeled with genomic *Ae. uniaristata* (N-genome, visualized in red) and *T. urartu* DNA (A-genome, visualized in green). Inserts: enlarged images showing the $2N^vS$ -2AS translocation. Arrowheads point to $2N^vS$ segments on the chromosome 2A of Jagger; arrows point to chromosomes 4A, which are known have an ancestral translocation between chromosome arms 4AL and 7BS. Bars, 10 µm.

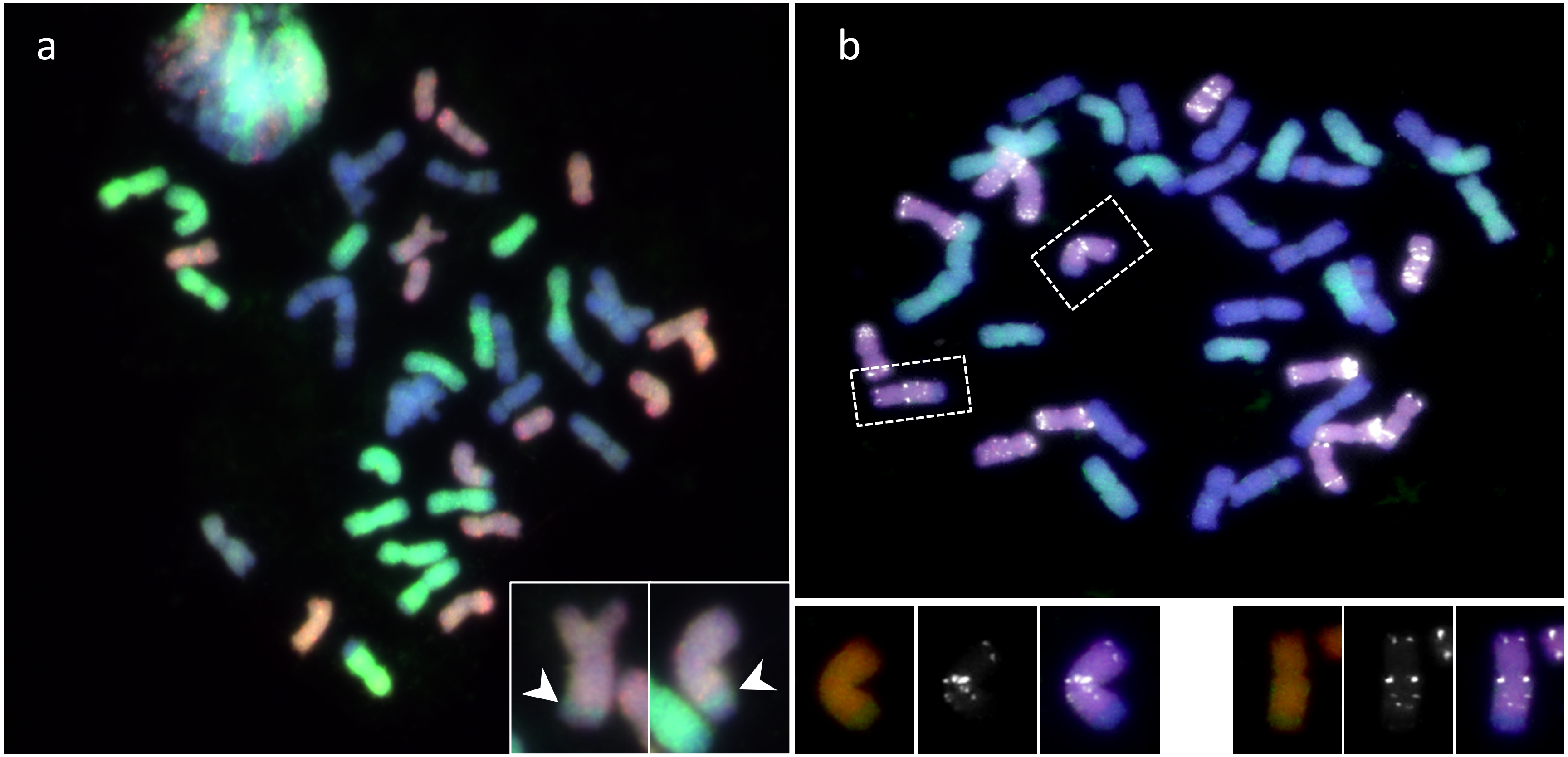

Supplementary Figure S2. GISH and sequential GISH-FISH patterns of 2DL inter-genomic translocation. (a) GISH pattern of mitotic metaphase chromosome of Jagger, labeled with genomic *Ae. tauschii* (D-genome, visualized in red) and *T. monococcum* DNA (A-genome, visualized in green). Inserts: enlarged images showing an intergenomic translocation involving 2DL and an unidentified A-genome chromosome. Arrowheads point to the A-genome segments located on interstitial regions of 2DL of Jagger; arrows point to chromosomes 4A. (b) Sequential GISH-FISH patterns of mitotic metaphase chromosomes of Jagger, labeled with genomic *Ae. tauschii* (D-genome, visualized in red), *T. urartu* DNA (A-genome, visualized in green) and pAS1 repeats (visualized in white). Inserts: enlarged images showing an intergenomic translocation involving 2DL and an unidentified A-genome chromosome.

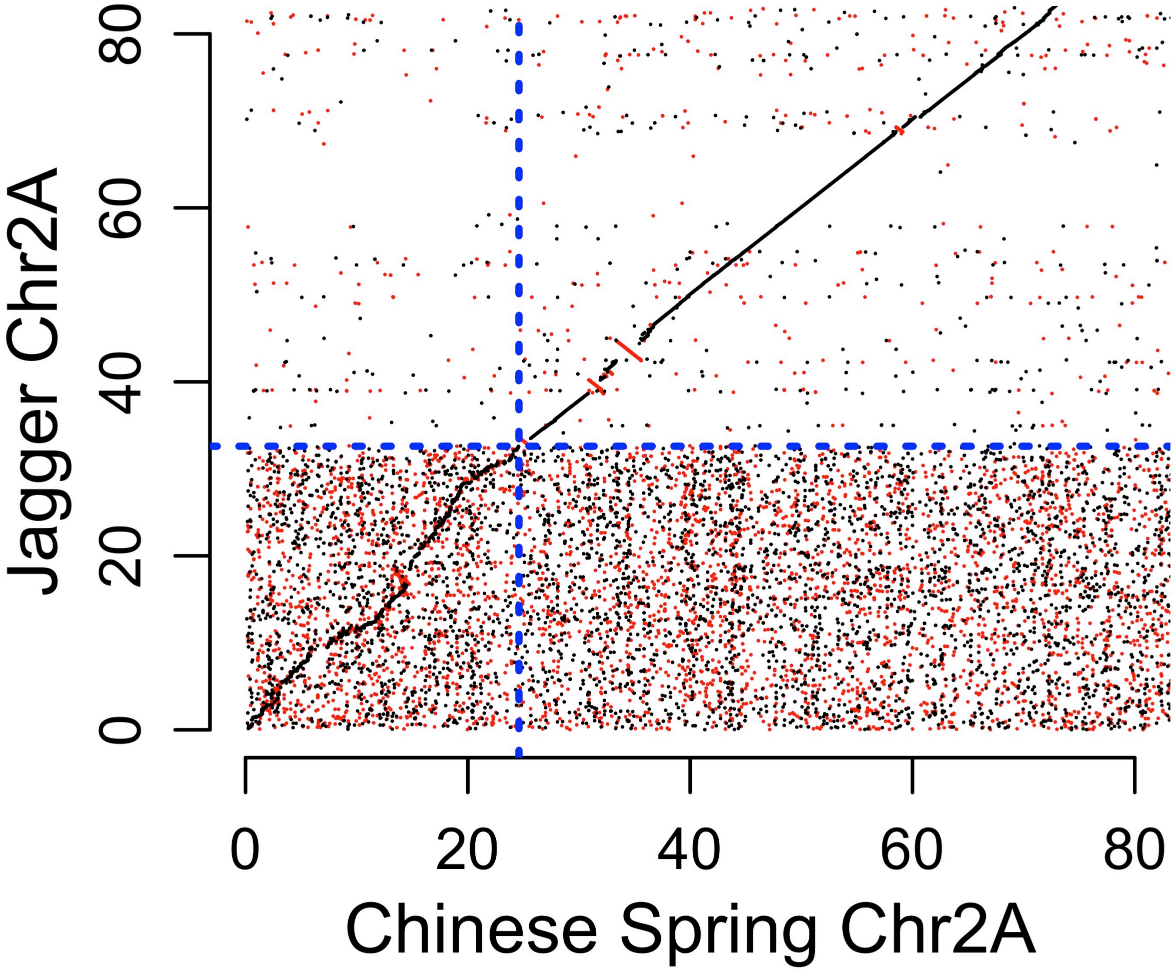

Supplemental Figure S3. Dotplot alignment of Jagger and Chinese Spring chromosome 2A. Figure showing all alignments regardless of percent identity or minimum alignment length.

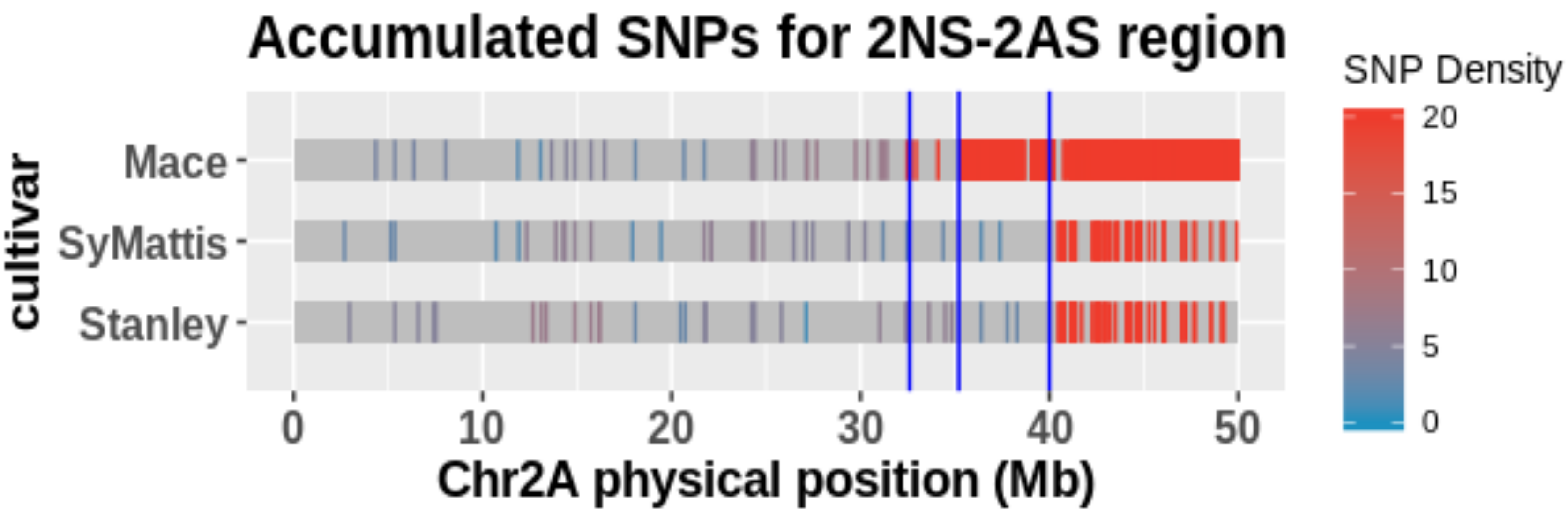

Supplemental Figure S4. Delineation of VPM1 segment in three potential 2NS carrier lines. The three blue vertical bars denote 32.6Mb ($2N^vS$ segment), 35Mb (reduction of VPM1 translocation) and 40Mb. There are relatively fewer SNPs in the ventricosa 2NS region (0-32.6Mb), and higher density of SNPs in the free-recombining wheat region (40Mb+). SNP density are based on number of SNPs per 5Mb region, with ceiling density set at 20.

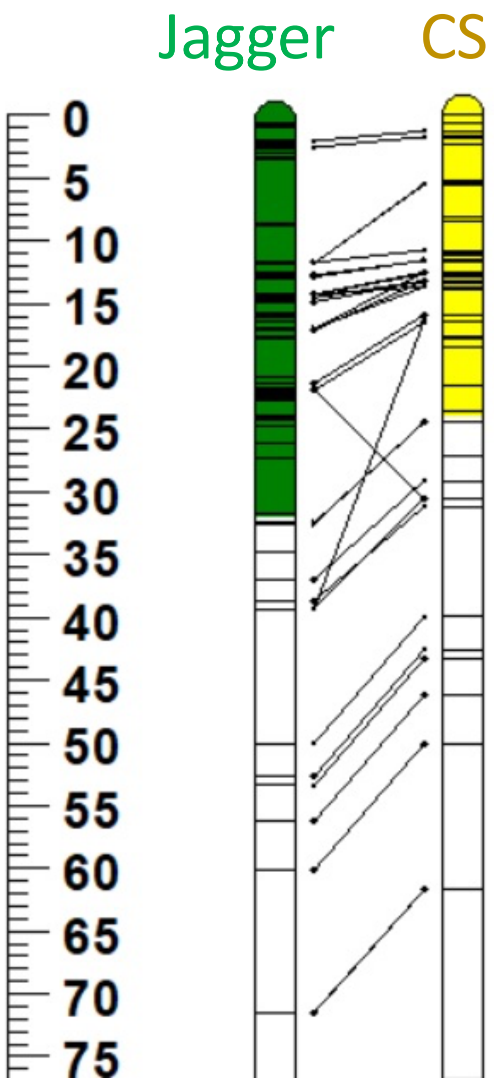

Supplemental Figure S5. Jagger chr2A NLR genes compared to Chinese Spring chr2A NLRs. The comparison is based on NLR annotator pipeline. Links between Jagger and CS indicate genes with the same motif structures. There is a lack of complete overlap of motif structures for the 2NS (green) NLRs and corresponding 2AS (dark blue) NLRs. Also, the number of 2NS NLRs is greater than the number of 2AS NLRs (black strokes on highlighted segments). Ruler indicates physical position (Mb).

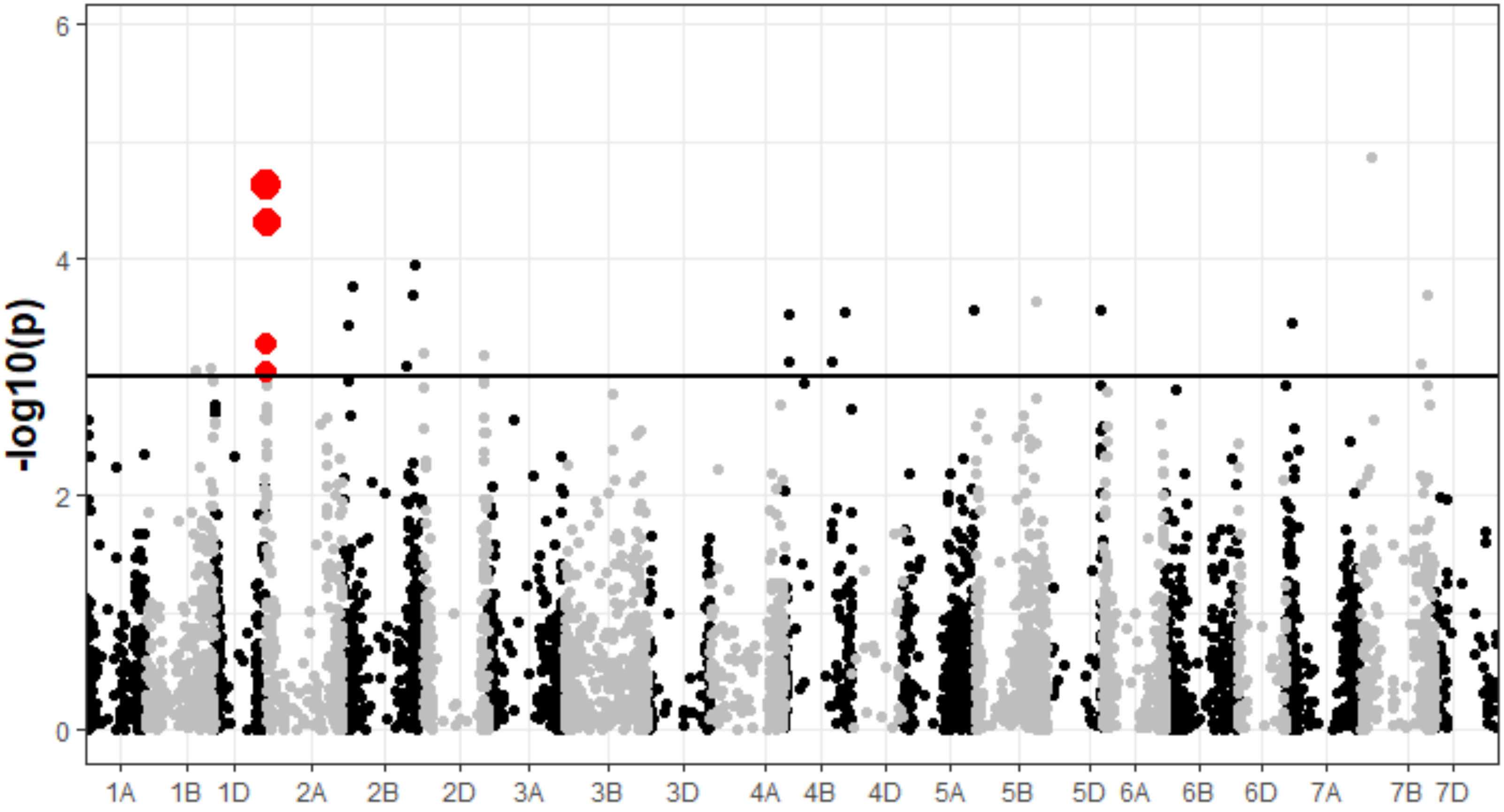

Supplemental Figure S6. Genome wide association study Manhattan plot based on USDA-RPN data, showing the significance of $2N^vS$ on wheat grain yield
